# Supplementary material for: New and sex-specific migraine susceptibility loci identified from a multiethnic genome-wide meta-analysis
Source: Commun Biol. 2021 Jul 22;4:864. doi: 10.1038/s42003-021-02356-y (PMC8298472; doi:10.1038/s42003-021-02356-y)
Supplement: Supplementary file 2 — Supplementary Information [file 42003_2021_2356_MOESM2_ESM.pdf]

Choquet et al. "New and sex-specific migraine susceptibility loci identified from a multiethnic genome-wide meta-analysis"

Supplementary Figures

Supplementary Figure 1. QQ plot and genomic inflation factors (λ) observed for the combined (GERA+UKB) multiethnic meta-analysis of migraine

Supplementary Figure 2. Correlation of effect sizes for migraine between GERA and UKB cohorts for the lead 22 migraine-associated lead SNPs identified in the combined (GERA+UKB) multiethnic meta-analysis

Supplementary Figure 3. LocusZoom plots of regions identified in the sex-specific (GERA+UKB) analyses that show differential association with migraine across women and men

Supplementary Figure 4. FUMA tissue eQTL specificity analysis for migraine-associated loci

Supplementary Figure 5. Genetic correlation between migraine and other diseases/traits

Supplementary Figure 6. Chicago plots of the multiethnic GWAS meta-analyses of migraine using PLINK vs. REGENIE.

Supplementary Figure 1. QQ plot and genomic inflation factors (λ) observed for the combined (GERA+UKB) multiethnic meta-analysis of migraine

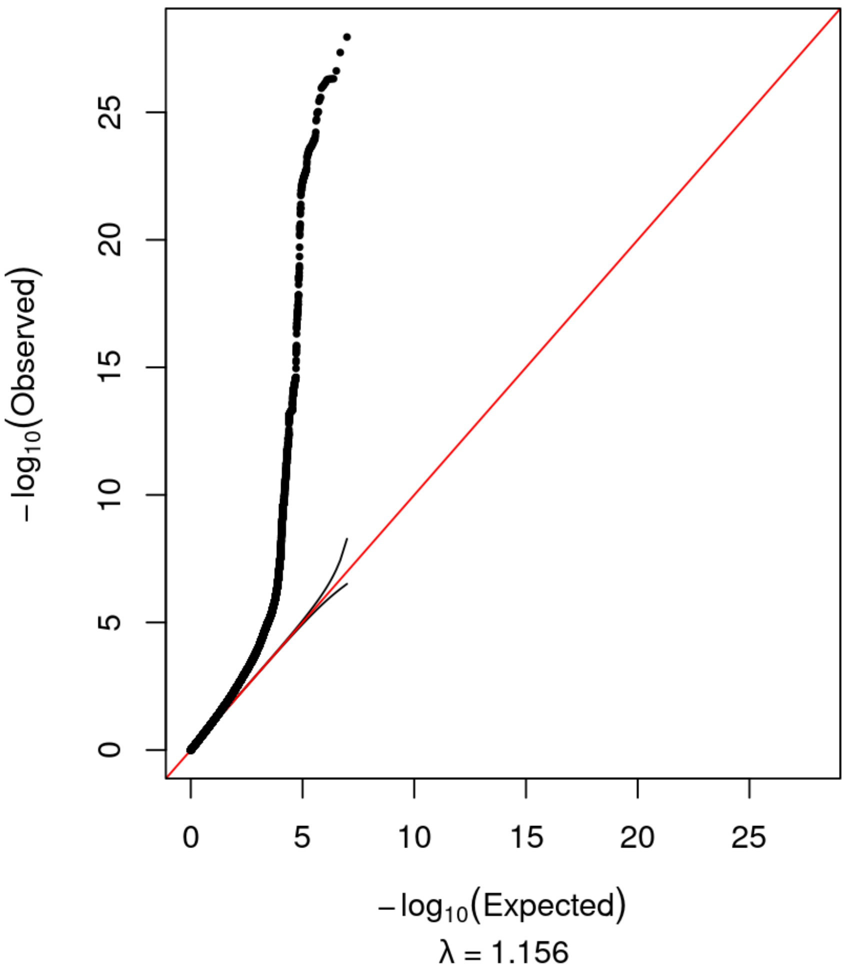

Supplementary Figure 2. Correlation of effect sizes for migraine between GERA and UKB cohorts for the lead 22 migraine-associated lead SNPs identified in the combined (GERA+UKB) multiethnic meta-analysis

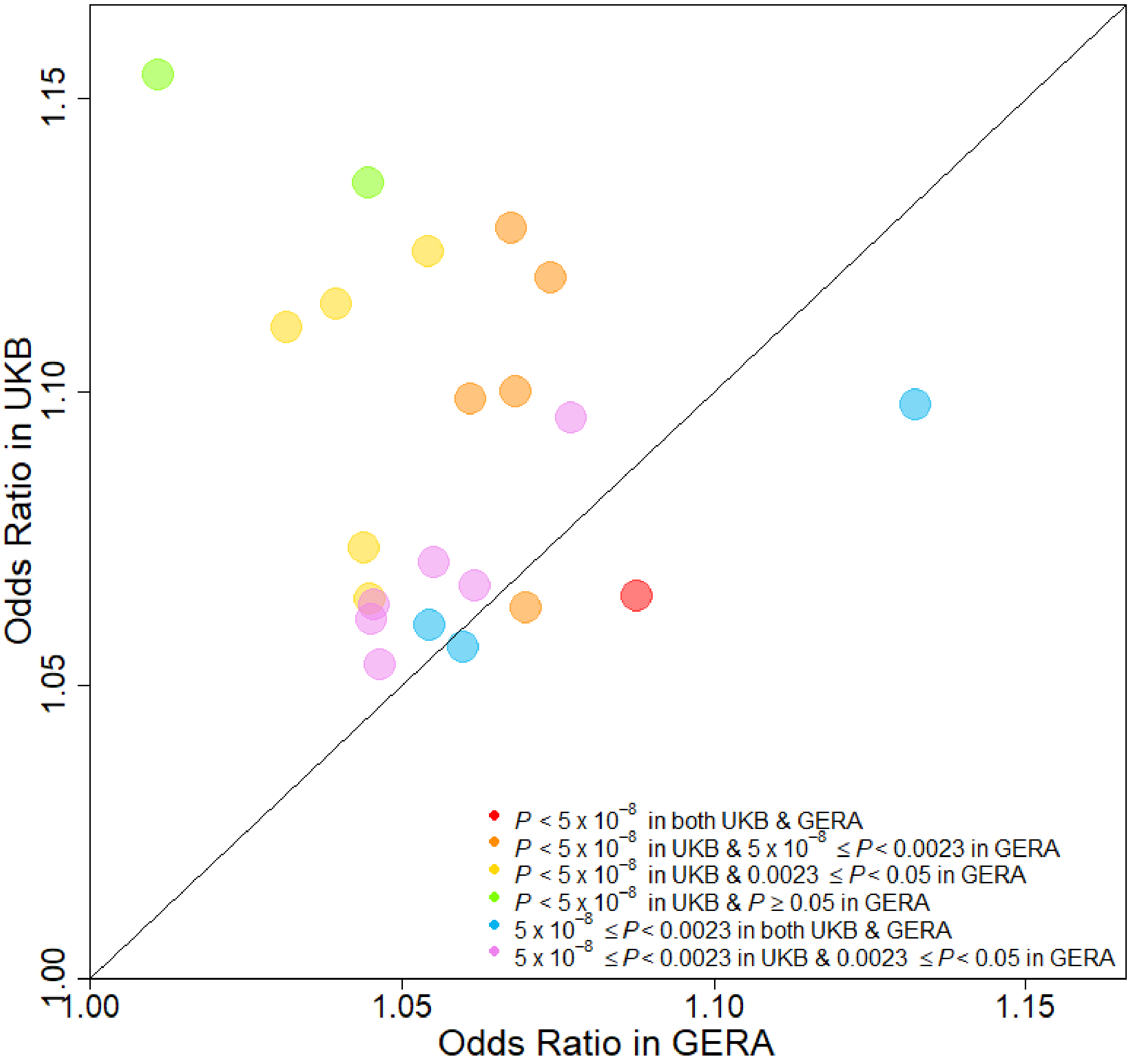

Supplementary Figure 3. LocusZoom plots of regions identified in the sex-specific (GERA+UKB) analyses that show differential association with migraine across women and men. The following regions were significant in women ($P<5x10^{-8}$) but not significant ($P>0.05$) in men: **a.** *CPS1*, **b.** *PBRM1*, **c.** *SLC25A21*; and **d.** *ASTN2*.

a.

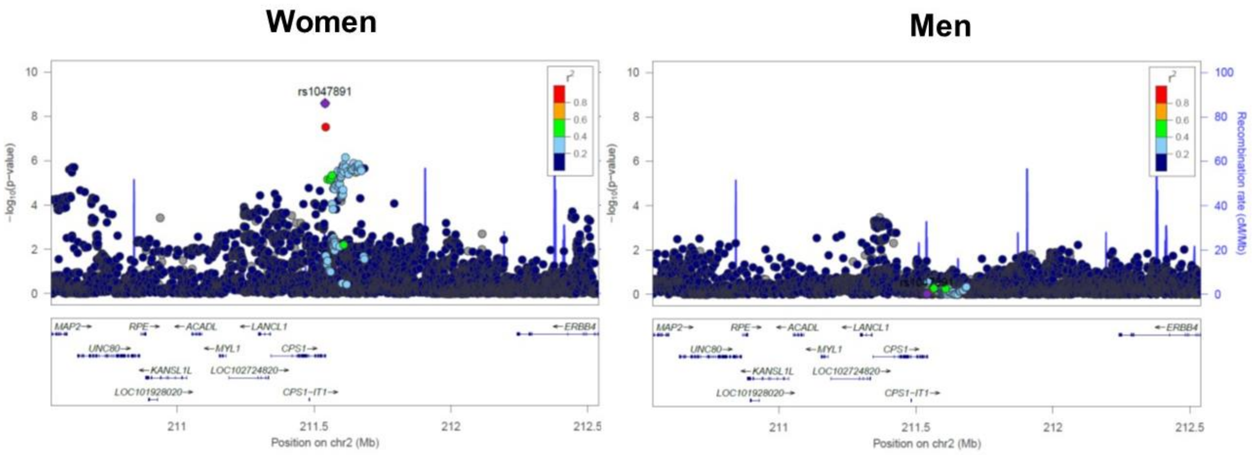

b.

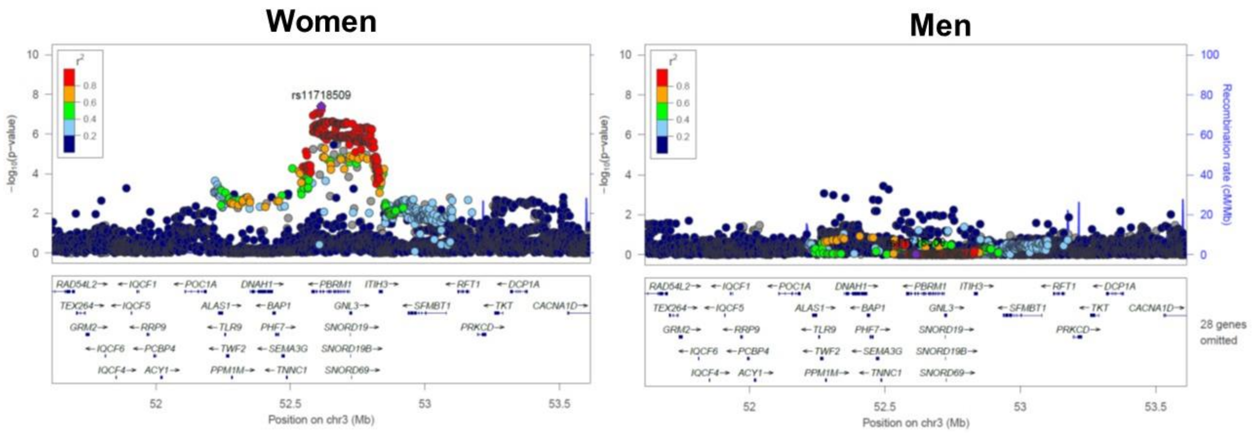

c.

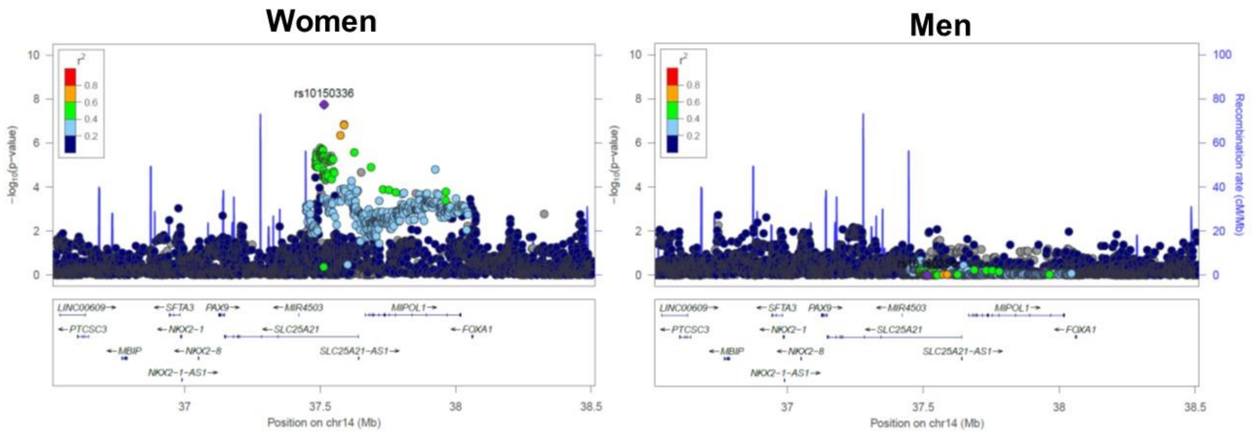
Women
Men
rs10150336
r²
0.8
0.6
0.4
0.2
−log10(p-value)
Recombination rate (cM/Mb)
LINC00609→
←SFTA3
PAX9→
←MIR4503
MIPOL1→
←PTCSC3
←NKX2-1
←SLC25A21
←FOXA1
←MBIP
←NKX2-8
SLC25A21-AS1→
NKX2-1-AS1→
Position on chr14 (Mb)

d.

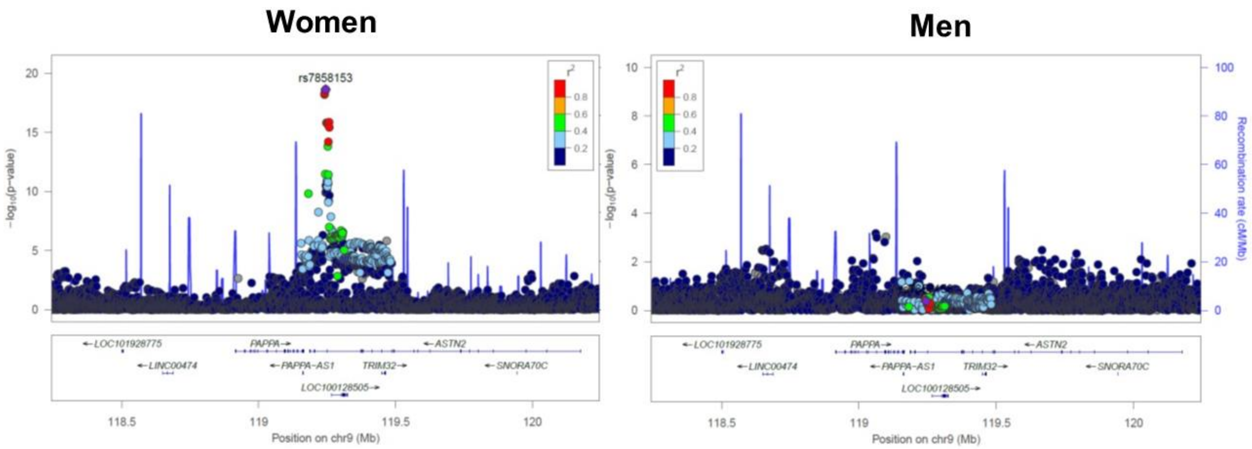
Women
Men
rs7858153
r²
0.8
0.6
0.4
0.2
−log10(p-value)
Recombination rate (cM/Mb)
←LOC101928775
PAPPA→
←ASTN2
←LINC00474
←PAPPA-AS1
TRIM32→
←SNORA70C
LOC100128505→
Position on chr9 (Mb)

Supplementary Figure 4. FUMA tissue eQTL specificity analysis for migraine-associated loci. As 53 GTEx tissues were tested, the P-value adjusted for Bonferroni correction was set as $P<9.43\times10^{-4}$ (0.05/53); GTEx tissues that reached this Bonferroni-level of significance are indicated as red bars.

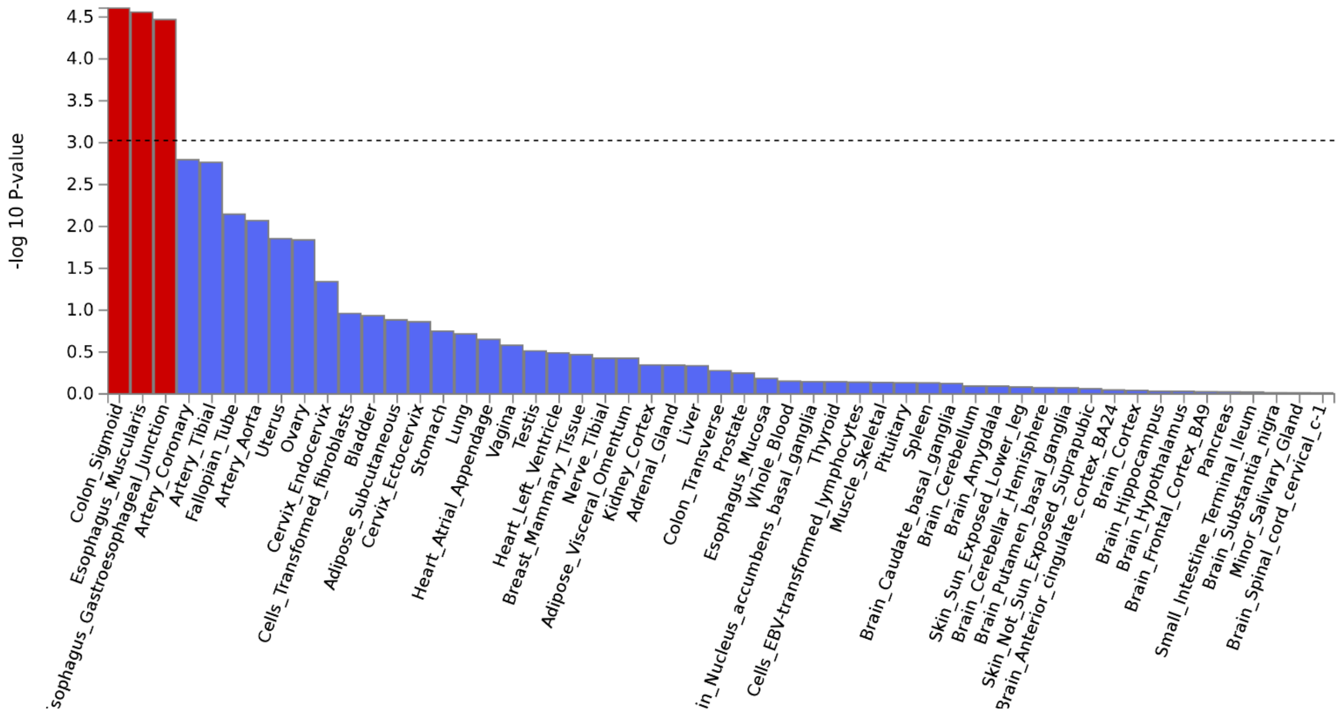

Supplementary Figure 5. Genetic correlation between migraine and other diseases/traits.

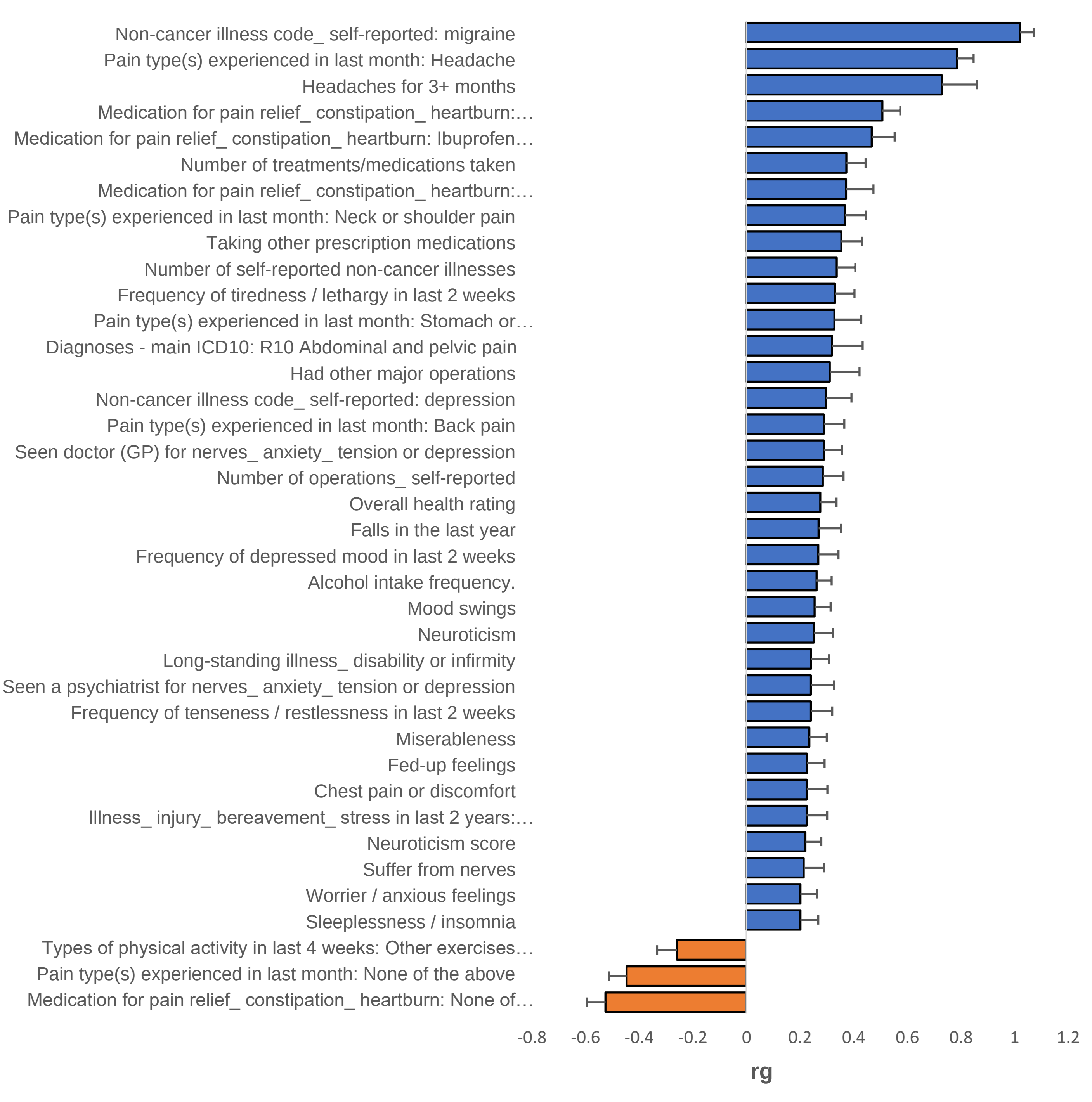

The results shown are those from an LD Score regression of the migraine meta-analysis (combining GERA non-Hispanic whites and UKB Europeans) and publicly available GWAS summary statistics (for N=772 diseases/traits at the time of writing). Only entries that reached a genome-level of significance ($P<5.0\times10^{-8}$) are shown in this figure.

Supplementary Figure 6. Chicago plots of the multiethnic GWAS meta-analyses of migraine using PLINK vs. REGENIE. Results from the meta-analyses generated using PLINK are presented on upper panel, while results from the meta-analysis generated using REGENIE are presented on the lower panel: **a.** Multiethnic GERA meta-analysis results, **b.** Multiethnic UKB meta-analysis results, and **c.** Overall (GERA+UKB) meta-analysis results. The y-axis represents the $-\log_{10}$(P-value). The red dotted line represents the threshold of P=5.0x10^{-8}.

a.

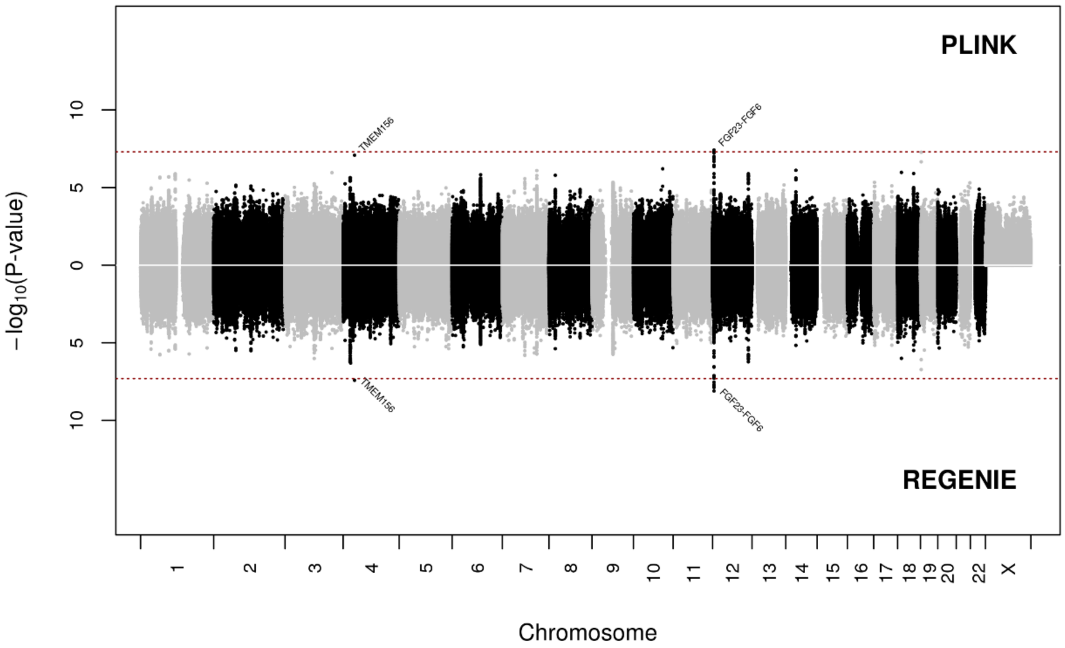

b.

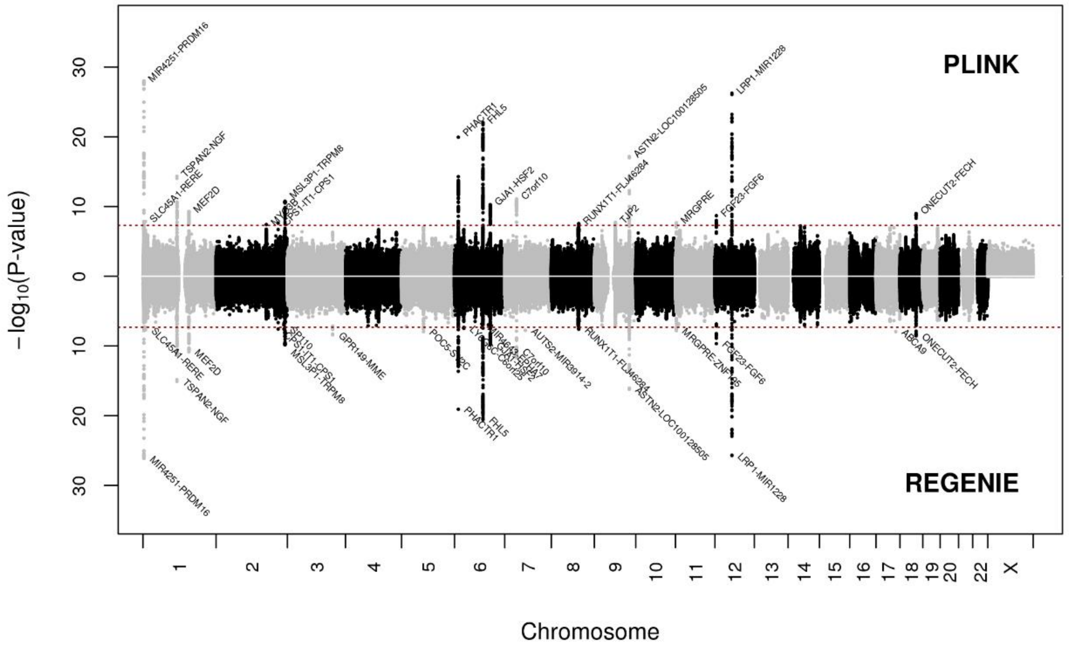

c.

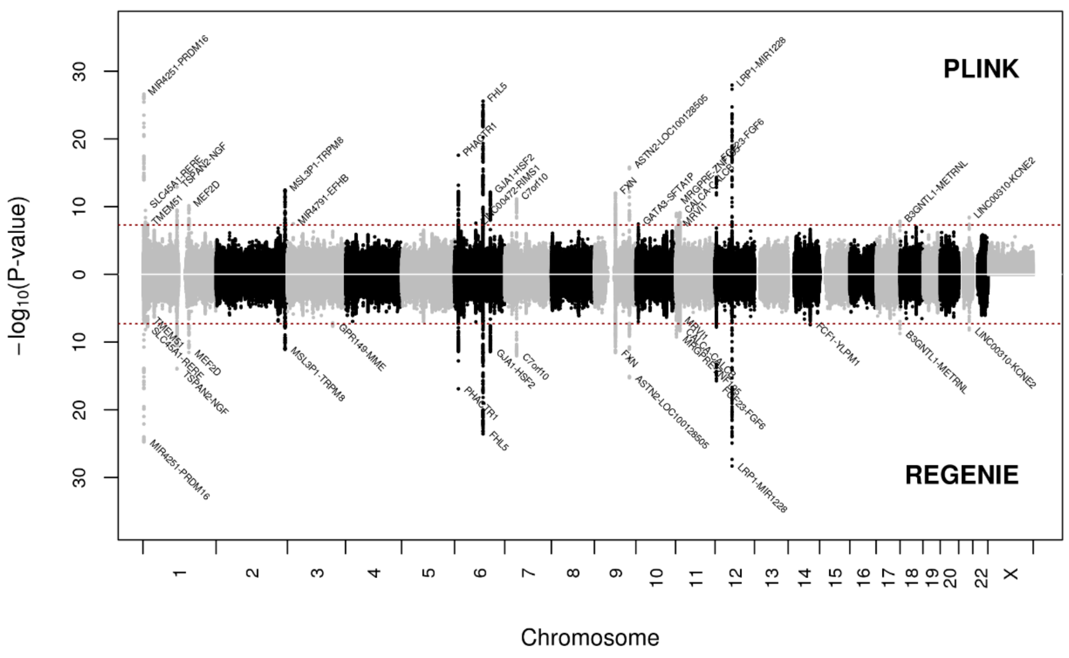
PLINK
REGENIE
−log10(P-value)
Chromosome
MIR4251-PRDM16
FHL5
PHACTR1
LRP1-MIR1228
FXN
ASTN2-LOC100128505
MSL3P1-TRPM8
GJA1-HSF2
C7orf10
MEF2D
TSPAN2-NGF
B3GNTL1-METRNL
LINC00310-KCNE2
GPR149-MME
FGF23-FGF6
GATA3-SFTA1P
TCF1-YLPM1
