# Supplementary material for: New and sex-specific migraine susceptibility loci identified from a multiethnic genome-wide meta-analysis
Source: Commun Biol. 2021 Jul 22;4:864. doi: 10.1038/s42003-021-02356-y (PMC8298472; doi:10.1038/s42003-021-02356-y)
Supplement: Supplementary file 3 — Description of Additional Supplementary Files [file 42003_2021_2356_MOESM3_ESM.pdf]

Description of Additional Supplementary Files

File name: Supplementary Data

Description:

Supplementary Data 1: Migraine loci identified in the combined (GERA-i-UKB) GWAS multiethnic meta-analysis and individual results in GERA and UKB.

Supplementary Data 2: Replication of Migraine loci identified in the combined (GERA-i-UKB) GWAS multiethnic meta-analysis in the IHGC (Gormley et al. Nature Genetics 2016).

Supplementary Data 3: Look-up of the 38 previously reported migraine loci in the combined (GERA-i-UKB) multiethnic analysis results.

Supplementary Data 4: Migraine-associated loci identified in the European ethnic-specific combined (GERA-i-UKB-i-IHGC) meta-analysis.

Supplementary Data 5: Migraine-associated loci identified in the women sex-specific combined (GERA-i-UKB) meta-analysis and look-up in men (GERA-i-UKB).

Supplementary Data 6: Comparison of the effect allele frequencies for the 4 women-specific lead SNPs between cases and controls in women and men.

Supplementary Data 7: Migraine-associated loci identified in the men sex-specific combined (GERA-i-UKB) meta-analysis and look-up in women (GERA-i-UKB).

Supplementary Data 8: List of the 95% credible set of variants in each of the 22 migraine loci identified in the combined (GERA-i-UKB) multiethnic analysis.

Supplementary Data 9: List of the 95% credible set of variants in each of the 73 migraine loci identified in the combined (GERA-i-UKB-i-IHGC) European analysis.

Supplementary Data 10: FUMA Gene-based association analysis top results for migraine.

Supplementary Data 11: DEPICT Gene Prioritization.

Supplementary Data 12: DEPICT Gene-set Enrichment.

Supplementary Data 13: FUMA Gene-set enrichment analysis.

Supplementary Data 14: FUMA tissue eQTL specificity analysis for migraine-associated loci.

Supplementary Data 15: DEPICT Tissue Enrichment.

Supplementary Data 16: Genetic risk sharing between migraine and other diseases/traits.

Supplementary Data 17: Migraine probability algorithm (MPA) used to define migraines cases and controls in GERA.

Supplementary Data 18: Comparison of the GWAS results generated using REGENIE versus PLINK.
