# Supplementary material for: New and sex-specific migraine susceptibility loci identified from a multiethnic genome-wide meta-analysis
Source: Commun Biol. 2021 Jul 22;4:864. doi: 10.1038/s42003-021-02356-y (PMC8298472; doi:10.1038/s42003-021-02356-y)
Supplement: Supplementary file 5 — Reporting Summary [file 42003_2021_2356_MOESM5_ESM.pdf]

nature research

Corresponding author(s): Hélène Choquet

Last updated by author(s): May 27, 2021

Reporting Summary

Nature Research wishes to improve the reproducibility of the work that we publish. This form provides structure for consistency and transparency in reporting. For further information on Nature Research policies, see our Editorial Policies and the Editorial Policy Checklist.

Statistics

For all statistical analyses, confirm that the following items are present in the figure legend, table legend, main text, or Methods section.

n/a	Confirmed	
☐	☒	The exact sample size (*n*) for each experimental group/condition, given as a discrete number and unit of measurement
☐	☒	A statement on whether measurements were taken from distinct samples or whether the same sample was measured repeatedly
☐	☒	The statistical test(s) used AND whether they are one- or two-sided<br>*Only common tests should be described solely by name; describe more complex techniques in the Methods section.*
☐	☒	A description of all covariates tested
☐	☒	A description of any assumptions or corrections, such as tests of normality and adjustment for multiple comparisons
☐	☒	A full description of the statistical parameters including central tendency (e.g. means) or other basic estimates (e.g. regression coefficient) AND variation (e.g. standard deviation) or associated estimates of uncertainty (e.g. confidence intervals)
☐	☒	For null hypothesis testing, the test statistic (e.g. *F*, *t*, *r*) with confidence intervals, effect sizes, degrees of freedom and *P* value noted<br>*Give P values as exact values whenever suitable.*
☒	☐	For Bayesian analysis, information on the choice of priors and Markov chain Monte Carlo settings
☐	☒	For hierarchical and complex designs, identification of the appropriate level for tests and full reporting of outcomes
☐	☒	Estimates of effect sizes (e.g. Cohen's *d*, Pearson's *r*), indicating how they were calculated

Our web collection on statistics for biologists contains articles on many of the points above.

Software and code

Policy information about availability of computer code

Data collection	Genotype analysis, quality control, phasing, and imputation analysis were performed on samples from the GERA cohort using the following softwares: Genotyping Console™ Software (Affymetrix) v4.0 to perform genotype calling, quality control (QC) analysis, and sample or SNP filtering prior to downstream analysis. PLINK software v1.90 to perform additional QC analyses. Genotypes were then pre-phased with Eagle5 v2.3.2, and then imputed with Minimac36 v2.0.1. All software programs employed are available for public use and no custom code was employed.
Data analysis	Eigenstrat v4.2 was used to calculate the principal components (PCs) on each of the four GERA ethnic groups. PLINK v1.9 was used to perform a logistic regression of the outcome and each SNP. The GWAS analyses were also conducted using a new approach accounting for relatedness that fits a whole genome regression model, implemented in REGENIEv2.0.2 (https://rgcgithub.github.io/regenie/). Other statistic analyses and data management were performed in the language-and-environment R, version 3.6.0, using functions from the default libraries. Genome-wide Complex Trait Analysis (GCTA) integrative tool was used to conduct a multi-SNP-based conditional & joint association analysis (COJO). CAVIARBF was used to prioritize genetic variants within the identified genomic regions. To prioritize genes and biological pathways, and highlight gene-set and tissue/cell enrichments within the identified migraine-associated loci, we used the FUMA integrative tool (https://fuma.ctglab.nl/) and DEPICT integrative tool (https://data.broadinstitute.org/mpg/depict/index.html). The LD Hub web interface was used to estimate the genetic correlation of migraine with more than 700 diseases/traits.

For manuscripts utilizing custom algorithms or software that are central to the research but not yet described in published literature, software must be made available to editors and reviewers. We strongly encourage code deposition in a community repository (e.g. GitHub). See the Nature Research guidelines for submitting code & software for further information.

Data

Policy information about availability of data

All manuscripts must include a data availability statement. This statement should provide the following information, where applicable:

- Accession codes, unique identifiers, or web links for publicly available datasets
- A list of figures that have associated raw data
- A description of any restrictions on data availability

The GERA genotype data are available upon application to the KP Research Bank (https://researchbank.kaiserpermanente.org/). The combined multiethnic (GERA +UKB) meta-analysis GWAS summary statistics are available from the NHGRI-EBI GWAS Catalog (https://www.ebi.ac.uk/gwas/downloads/summary-statistics), study accession number GCST90000016. GWAS summary statistics data (SNPs with P<1.0x10-5) from the study of Gormley et al.21, are publicly accessible (http://eagle-i.itmat.upenn.edu/i/00000155-e1db-73aa-c956-e86e80000000).

Field-specific reporting

Please select the one below that is the best fit for your research. If you are not sure, read the appropriate sections before making your selection.

[x] Life sciences [ ] Behavioural & social sciences [ ] Ecological, evolutionary & environmental sciences

For a reference copy of the document with all sections, see nature.com/documents/nr-reporting-summary-flat.pdf

Life sciences study design

All studies must disclose on these points even when the disclosure is negative.

Sample size

In this study, we conducted a genome-wide association analyses, followed by meta-analysis, including 554,569 individuals (28,852 cases and 525,717 controls) from two cohorts: the Genetic Epidemiology Research in Adult Health and Aging (GERA) and the UK Biobank (UKB). Rather than performing a power calculation, we collected the largest possible GWAS for migraine to date to identify novel risk loci.

In GERA, patients with migraine were identified in the KPNC electronic health record system using a validated migraine probability algorithm (MPA), which is based on migraine-specific prescriptions and International Classification of Disease, Ninth (ICD9) diagnosis code: 346.XX and Tenth (ICD10): G43.XX. We defined migraine cases as patients with a score > 10 on the MPA (any evidence of migraine). After excluding individuals with any evidence of headache without a migraine diagnosis, as well as individuals with a MPA score = 10, our control group included all the non-cases. In total, 11,320 migraine cases and 60,282 controls from GERA were included in this study.

In UKB, migraine cases (N=17,532) were defined as participants with a self-reported migraine (data field 20002 code 1265) and/or a diagnosis code for migraine (ICD-10: G43). After excluding participants who self-reported headaches (data field 20002, code 1436) and/or who had a diagnosis code for headaches (ICD-10: G44), the control group included 465,435 participants who were not cases.

Data exclusions

In GERA, genotype quality control (QC) procedures and imputation were conducted on an array-wise basis, after an updated genotyping algorithm with an advanced normalization step specifically for SNPs in batches not recommended or flagged by the outlier plate detector than has previously been done. Subsequently, variants were excluded if: >3 clusters were identified; the number of batches was <38/42 (EUR array), <3/5 (AFR), <3/6 (EAS), or <7/9 (LAT); and the ratio of expected allele frequency variance across packages was <100 (EUR), <50 (AFR), <100 (EAS), <200 (LAT). On the EUR array, variants were additionally excluded if heterozygosity >.52 or <.02, and if an association test between Reagent kit v1.0 and v2.0 had P<10-4. Imputation was done by array, and we additionally removed variants with call rates <90%.

Replication

Replication analyses of the loci identified in the combined (GERA+UKB) meta-analysis as well as the loci identified through the sex-specific analyses were conducted using the GWAS summary statistics data from the study of Gormley et al., consisting of 375,752 participants (including 59,674 migraine cases) from the IHGC, which were publicly accessible.

Randomization

Samples were not randomized. This is a case-control study where cases were those with migraine, and controls were non-cases. Association analyses were adjusted for age, sex, and principal components as covariates.

In GERA, patients with migraine were identified in the KPNC electronic health record system using a validated migraine probability algorithm (MPA), which is based on migraine-specific prescriptions and International Classification of Disease, Ninth (ICD9) diagnosis code: 346.XX and Tenth (ICD10): G43.XX. We defined migraine cases as patients with a score > 10 on the MPA (any evidence of migraine). After excluding individuals with any evidence of headache without a migraine diagnosis, as well as individuals with a MPA score = 10, our control group included all the non-cases. In total, 11,320 migraine cases and 60,282 controls from GERA were included in this study.

In UKB, migraine cases (N=17,532) were defined as participants with a self-reported migraine (data field 20002 code 1265) and/or a diagnosis code for migraine (ICD-10: G43). After excluding participants who self-reported headaches (data field 20002, code 1436) and/or who had a diagnosis code for headaches (ICD-10: G44), the control group included 465,435 participants who were not cases.

Blinding

Blinding was not relevant to our study, since participants were selected based on their migraine status.

Reporting for specific materials, systems and methods

We require information from authors about some types of materials, experimental systems and methods used in many studies. Here, indicate whether each material, system or method listed is relevant to your study. If you are not sure if a list item applies to your research, read the appropriate section before selecting a response.

Materials & experimental systems

n/a	Involved in the study	
☒	☐	Antibodies
☒	☐	Eukaryotic cell lines
☒	☐	Palaeontology and archaeology
☒	☐	Animals and other organisms
☐	☒	Human research participants
☒	☐	Clinical data
☒	☐	Dual use research of concern

Methods

n/a	Involved in the study	
☒	☐	ChIP-seq
☒	☐	Flow cytometry
☒	☐	MRI-based neuroimaging

Human research participants

Policy information about studies involving human research participants

Population characteristics	The Genetic Epidemiology Research in Adult Health and Aging (GERA) cohort consists of 110,266 adult men and women, 18 years and older, who are of non-Hispanic white, Hispanic/Latino, Asian or African American ethnicity.<br>The UK Biobank (UKB) is a large prospective study following the health of approximately 500,000 participants from 5 ethnic groups (European, East Asian, South Asian, African British, and mixed ancestries) resident in the UK aged between 40 and 69 years-old at the baseline recruitment visit.
Recruitment	Participants from the GERA cohort are members of the Kaiser Permanente Northern California (KPNC) integrated health care delivery system, and provided self-reported information via the Research Program on Genes, Environment, and Health (RPGEH) survey.<br>For UKB participants, demographic information and medical history were ascertained through touch-screen questionnaires. UKB participants also underwent a wide range of physical and cognitive assessments, including blood sampling.<br>Potential Recruitment biases due to self-reports: the self-reported migraine in some UKB cases is unlikely to bias the results of this study as we observed a very high concordance between the GWAS results for migraine validated cases compared to self-reported in UKB. Further, most of our results were replicated in the International Headache Genetics Consortium (IHGC) data.
Ethics oversight	For GERA, all study procedures were approved by the Institutional Review Board of the Kaiser Permanente Northern California Institutional Review Board. Written informed consent was obtained from all participants.<br>For UKB, the analyses presented in this paper were carried out under UK Biobank Resource project #14105.

Note that full information on the approval of the study protocol must also be provided in the manuscript.
